# Familiar objects benefit more from transsaccadic feature predictions

**DOI:** 10.3758/s13414-022-02651-8

**Published:** 2023-01-31

**Authors:** Nedim Goktepe, Alexander C. Schütz

**Affiliations:** 1 AG Allgemeine und Biologische Psychologie, Philipps-Universität Marburg, Marburg, Germany; 2 Center for Mind, Brain and Behavior, Philipps-Universität Marburg, Marburg, Germany

**Keywords:** Saccades, Transsaccadic prediction, Perceptual learning, Object recognition, Visual stability

## Abstract

The transsaccadic feature prediction mechanism associates peripheral and foveal information belonging to the same object to make predictions about how an object seen in the periphery would appear in the fovea or vice versa. It is unclear if such transsaccadic predictions require experience with the object such that only familiar objects benefit from this mechanism by virtue of having peripheral-foveal associations. In two experiments, we tested whether familiar objects have an advantage over novel objects in peripheral-foveal matching and transsaccadic change detection tasks. In both experiments, observers were unknowingly familiarized with a small set of stimuli by completing a sham orientation change detection task. In the first experiment, observers subsequently performed a peripheral-foveal matching task, where they needed to pick the foveal test object that matched a briefly presented peripheral target. In the second experiment, observers subsequently performed a transsaccadic object change detection task where a peripheral target was exchanged or not exchanged with another target after the saccade, either immediately or after a 300-ms blank period. We found an advantage of familiar objects over novel objects in both experiments. While foveal-peripheral associations explained the familiarity effect in the matching task of the first experiment, the second experiment provided evidence for the advantage of peripheral-foveal associations in transsaccadic object change detection. Introducing a postsaccadic blank improved change detection performance in general but more for familiar than for novel objects. We conclude that familiar objects benefit from additional object-specific predictions.

## Introduction

The visual system on average executes three to four saccades per second to reposition the fovea to sample high-resolution information about objects or locations of interest in the visual scene (Rayner, 1998). Thus, by bringing an object of interest to the fovea, it samples information at two different resolutions about that object: Prior to a saccade, the visual system samples information in low resolution with peripheral vision. When gaze is shifted to the target object, it samples high-resolution information with foveal vision. This leads to the fundamental question about how the visual system achieves perceptual stability despite the large differences in object information from the peripheral and foveal visual field (for reviews, see Mathôt & Theeuwes, 2011; Melcher, 2011; Rolfs, 2015; Stewart et al., 2020; Wurtz, 2008; Wurtz, 2018). Different mechanisms might contribute to the seamless perception across saccades.

Recent evidence suggests that the visual system can optimally integrate peripheral and foveal information across saccades (e.g., Ganmor et al., 2015; Hübner & Schütz, 2017; Stewart & Schütz, 2019a; Wolf & Schütz, 2015; for a review, see Stewart et al., 2020). However, it is not always adequate or possible to integrate peripheral and foveal information. When the sampled information from the periphery and the fovea is discrepant (e.g., when the saccade target is displaced or exchanged with another object), the visual system can segregate pre- and postsaccadic information (e.g., Atsma et al., 2016; Demeyer et al., 2010; Laurin et al., 2021; Tas et al., 2012; Tas et al., 2021). Furthermore, in some cases, the visual system samples object information with only peripheral vision (Treisman, 1986), and visual search is surprisingly unaffected by blocking foveal vision (David et al., 2021; Nuthmann, 2014; Nuthmann & Canas-Bajo, 2022). According to the transsaccadic feature prediction mechanism (Herwig & Schneider, 2014), object recognition and visual search is supported by predictions based on previous associations of peripheral and foveal information of objects. The associated information serves as a base of prediction of object features and identities. Herwig and Schneider (2014) argued that prediction from the associated information works bi-directionally: While peripheral object information is used to predict the foveal information of peripheral objects (peripheral object recognition), in return, foveal object information is used to predict the peripheral information of the same object to facilitate visual search. Thus, when peripheral and foveal information of an object is associated, recognizing the said object in the periphery is facilitated through sampled peripheral information and the associated foveal prediction (i.e., peripheral-foveal prediction). Similarly, when the same object becomes the visual search target, the available foveal information and the associated peripheral prediction facilitates visual search (i.e., foveal-peripheral prediction). They provided direct evidence for their account in two experiments in which object features were manipulated across eye movements. Manipulation of peripheral-foveal associations by means of swapping pre- and postsaccadic objects in training shifted subsequent peripheral judgments of these objects towards to the swapped object, quantifying the role of transsaccadic prediction for trained objects. Studies using similar designs provided evidence that the transsaccadic feature prediction mechanism operates with simple visual features such as shapes (Herwig et al., 2015) as well as complex stimuli such as faces and everyday objects (Osterbrink & Herwig, 2021). Furthermore, Köller et al. (2020) showed that the contribution of transsaccadic feature predictions is modulated by the discrepancy between the associated peripheral and foveal information. Hence, transsaccadic predictions could be another factor to consider for understanding the transsaccadic integration puzzle (Herwig, 2015).

Previous studies corroborated evidence that the visual system uses previously associated peripheral and foveal information belonging to the same object to make predictions and to facilitate visual tasks. However, in daily life we are surrounded by a myriad of objects, some of which are familiar (i.e., previously encountered objects), while others are completely novel. With its more limited peripheral vision (for reviews, see Rosenholtz, 2016; Strasburger, 2020; Strasburger et al., 2011; Whitney & Levi, 2011), the visual system is tasked with identifying saccade targets to quickly and efficiently sample information (e.g., Najemnik & Geisler, 2005; for a review, see Eckstein, 2011). Therefore, an ecologically important question is whether transsaccadic predictions are limited to familiar objects or whether they also apply to novel objects. While object-specific predictions for familiar objects can be made based on previously acquired transsaccadic associations as demonstrated by Herwig and Schneider (2014), novel objects that lack these object-specific predictions could generate predictions based on experience in transsaccadic changes (i.e., how visual appearance typically changes at different eccentricities). Hence, object predictions could be conceptualized as a ladder where at the lower end predictions are made for novel objects solely based on coarse expectations of transsaccadic changes. However, at the upper end of the ladder, predictions could be based on more precise object-specific predictions available for familiar objects. Hence, one would expect the precision and accuracy of object predictions to increase with increased familiarity. Previous studies compared visual search and peripheral recognition performance of objects that are swapped or not swapped with another object during training to measure transsaccadic prediction effects (Cox et al., 2005; Herwig et al., 2018; Herwig & Schneider, 2014; Köller et al., 2020; Osterbrink & Herwig, 2021; Paeye et al., 2018; Valsecchi et al., 2020; Valsecchi & Gegenfurtner, 2016). Therefore, observers were equally familiar with both classes of objects during the test phase where the effect of transsaccadic prediction was measured. Thus, whether object-specific predictions from previous transsaccadic associations provide an advantage to familiar objects compared to novel objects with coarse predictions remains unanswered.

Another important question for the everyday relevance of the transsaccadic prediction mechanism is the specificity of the peripheral-foveal predictions. Do these associations reflect a generative mechanism translating between peripheral and foveal representations independent of location and object or do they reflect rather narrow object- and location-specific associations? Two studies (Valsecchi et al., 2020; Valsecchi & Gegenfurtner, 2016) provided evidence that the visual system uses peripheral-foveal associations of object size for predicting the size of objects at the same as well as at the opposite hemifield. In contrast, Herwig et al. (2018) reported that the use of peripheral-foveal associations in prediction is restricted to the location of learning. However, it is important to note the differences between the two groups of studies. First, while Valsecchi and Gegenfurtner (2016) and Valsecchi et al. (2020) manipulated object size, Herwig et al. (2018) manipulated spatial frequency of the transsaccadic objects. Other than being different visual features, perceived size is expected to be uniformly changing across symmetrical eccentricities at opposite hemifields. On the other hand, even if perceived spatial frequency changes symmetrically across visual hemifields, Herwig et al. (2018) did not use symmetric locations to test location specificity. Thus, the location specificity of object predictions is an open question.

To address these questions, we conducted two experiments. Since familiarity with an object varies with individual experience, we devised a training phase where observers unknowingly familiarized with a set of novel objects by performing a sham task. In the first experiment, we manipulated available foveal and peripheral information to test if peripheral-foveal or foveal-peripheral predictions provide an advantage to familiar compared to novel objects. Due to the design of the first experiment, observers did not execute saccades. However, in daily life we constantly execute saccades to sample information. Therefore, saccades are also important for understanding the contribution of object-specific predictions for familiar objects. We addressed this in the second experiment, by testing whether having object-specific predictions for familiar objects improves transsaccadic object change detection compared to novel objects that lack object-specific predictions.

## Experiment 1: Peripheral-foveal object matches

**Fig. 1:**
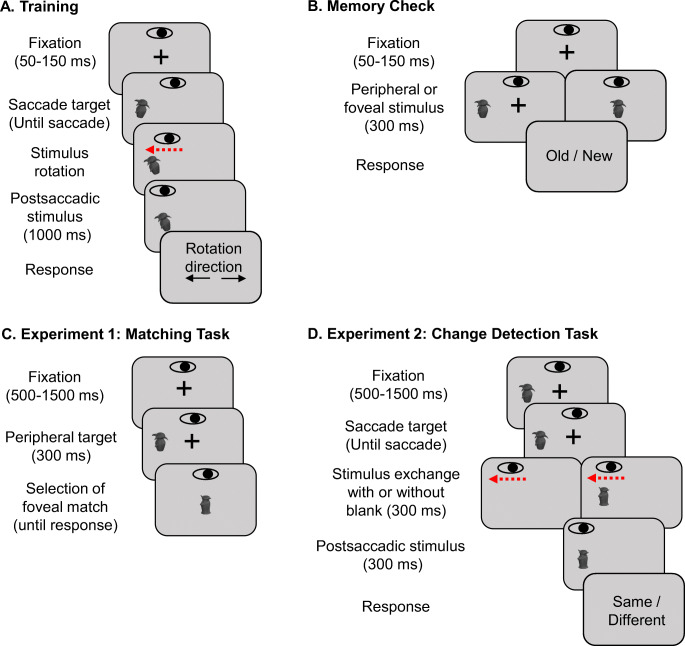
Schematic illustration of trials in training (**A**), memory check (**B**), and the experimental task in Experiments 1 and 2 (**C, D**). Observers performed the same training and memory check on day 1, then completed Experiment 1 or Experiment 2 on the following day. For illustration purposes, stimulus size and rotation are exaggerated. The response screen was blank in the experiments. The eye icon represents horizontal gaze position and is vertically offset for illustration purposes

Based on the transsaccadic feature mechanism, object-specific predictions stemming from previously associated peripheral and foveal information should provide additional advantage compared to novel objects that lack such predictions. One premise of the transsaccadic feature prediction mechanism is that object-specific transsaccadic associations improve the matching of peripheral and foveal information. To test this hypothesis, we asked observers to match peripheral and foveal objects in a 3-AFC (three-alternative forced choice) matching task (Fig. 1).

An important aspect of the peripheral-foveal association is the assumption that those associations could be used to make bi-directional predictions (Herwig & Schneider, 2014). That means peripheral sensory information could predict foveal information (peripheral-foveal prediction) and foveal sensory information could predict peripheral information (foveal-peripheral prediction). Therefore, the familiarity of both peripheral targets as well as foveal options could affect the matching performance. On one hand, familiar peripheral targets could provide object-specific peripheral-foveal predictions that provide a direct comparison for foveal information for the different response options. On the other hand, familiar foveal options could benefit the matching of both familiar and novel peripheral targets by providing specific foveal-peripheral predictions. Therefore, we systematically manipulated the number of familiar options to test how the bi-directional predictions contribute to peripheral-foveal matching. We would expect better performance in trials with familiar than novel peripheral targets if observers make use of peripheral-foveal predictions. Similarly, we would expect to see performance to improve with increasing number of familiar foveal response options, if foveal-peripheral predictions are used by observers.

### Methods

#### Observers

In total, 15 observers (11 females, mean age = 24.33 ± 4.29 years) with normal or corrected-to-normal vision participated in the experiment. Observers were university students; they signed an informed consent form and were compensated with 8 euros per hour. All experiments were approved by the local ethics commission of the Department of Psychology of Marburg University (proposal number 2015-35k) and were conducted in accordance with the Declaration of Helsinki (1964).

#### Stimuli and apparatus

Stimuli were taken from greebles stimulus set (tarrlab.com). Greebles are systematically varying humanlike objects that have been used extensively in psychological research (Gauthier & Tarr, 1997; Gauthier et al., 1999). We chose to use greebles since they systematically capture similarities (e.g., shape of an orange and a tennis ball) as well as differences (e.g., color of an orange and a tennis ball) between everyday objects. Each greeble has a gender (male or female), a family (one of four family body features), and a family trait (one of four body parts). All greebles were rendered achromatic using MATLAB’s *rgb2gray* function. We chose four greebles to be familiarized during the training phase. These greebles only shared one of their features with any other greeble in the same set. Therefore, each familiar greeble had two different features compared to the rest. Similarly, the novel greebles were selected to share only one feature with all other greebles including familiar greebles. Greebles subtended approximately an area of 3 × 5 visual degrees on a neutral gray background.

Throughout the experiments, the stimuli were presented at a 106-cm viewing distance on a back-projection setup, including a PROPixx projector (VPixx Technologies, Saint Bruno, QC, Canada), with a resolution of 1,920 × 1,080 pixels at 120 Hz and a 91 × 51-cm screen from Stewart Filmscreen (Torrance, CA, USA). The display was gamma corrected for linear luminance output and calibrated to minimize the central hot spot. After calibration, it had a luminance of 2.07, 71, and 140 cd/m^2^ for black, gray, and white pixels, respectively. We used MATLAB (MathWorks Inc., Natick, MA, USA) along with Psychtoolbox (Brainard, 1997; Kleiner, Brainard, & Pelli, 2007) and EyelinkToolbox (Cornelissen et al., 2002) for programing and running the experiments. Eye movements were recorded with an Eyelink 1000+ eye tracker (SR Research Ltd., Kanata, ON, Canada) at 1,000-Hz sampling rate. Eye-tracker calibration was done using the right eye of the observers with a 9-point grid. Fixation points on the grid were placed around the center of the display at 10° horizontal and 5° vertical eccentricities. A fixation check was carried out at the beginning of each trial. The eye tracker was re-calibrated after half of the trials were completed. The heads of the observers were stabilized using a chin and forehead rest.

#### Procedure

##### Training phase

The training consisted of a transsaccadic orientation change detection task using a set of four Greebles chosen for familiarization. The observer’s task was to make a saccade to the peripheral object and report the direction of the orientation change. In each trial, after a random fixation period of 50–150 ms, one of the four greebles was randomly selected and presented at 10° left of the fixation target. The peripheral object had a random orientation between -45 and 45°. The peripheral object was tilted by ± 5° when the measured eye position deviated more than 2° away from the fixation target. The now foveated object stayed on the screen for 1,000 ms before it was extinguished. Observers reported the direction of the rotation by using the left and right arrow keys. The experiment consisted of 640 trials in total (160 trials per greeble) and took about 1.5 h.

##### Memory check

On the following day, observers first completed a recognition task to check their memory of the greebles. Four greebles from training and four novel greebles differing in one dimension were used. Observers were asked to indicate whether they recognized the presented greeble from the previous day. In the first half of the trials, greebles were presented peripherally at 10° left or right of the center of the display. In the second half, greebles were presented foveally at the center of the display. The greeble was extinguished if the gaze position was outside of an imaginary 4°× 7° square central to display. The greeble reappeared when gaze returned to the imaginary square. Greebles were presented for a total duration of 300 ms in five blocks for each viewing condition.

##### Test phase

Following the memory check, observers proceeded with the 3-AFC peripheral-foveal matching task. Each trial started with a fixation check followed by a central fixation cross. After a random period of 500–1,500 ms, a novel or familiar greeble was displayed for a total of 300 ms at 10° either left or right of the fixation with equal probability. To ensure peripheral presentation, the greeble (*peripheral target* onwards) was extinguished when the measured gaze position was more than 2° away from the fixation. At the end of the peripheral target presentation, one of three options (one matching the peripheral target and two different greebles) was randomly presented in the center of the screen at fixation. The non-matching options always shared two out of three features with the peripheral target and were systematically varied to be both familiar, both novel, or one of each. Therefore, for novel peripheral targets, the overall number of familiar response options varied between zero and two. For familiar peripheral targets, the overall number of familiar response options varied between one and three. Observers were instructed to scroll through the options by using up and down arrows until they identified the target. To make sure that only foveal information was presented to the observers during the response period, the current option was replaced by a noise mask when the gaze deviated more than 2° from the center. In addition, to avoid potential aftereffects from quickly scrolling through options, a central noise mask was presented for 300 ms each time the observers scrolled to another option. The experiment consisted of 240 trials (2 (peripheral target familiarity: familiar, novel) × 2 (target location: left, right) × 3 (number of familiar options: 0, 1, 2 for novel peripheral targets; 1, 2, 3 for familiar peripheral targets) × 20 repetitions).

#### Data analysis

We intended to use the memory check as a control measure for familiarization training. To assess if the memory check was an adequate measure of observers’ familiarity, we checked the relationship between the performance in the memory check and in the matching task of Experiment 1. We correlated the memory performance with the difference between all novel and all familiar conditions, which reflects the strongest familiarity contrast across conditions. There was no significant relationship between memory check performance and familiarity-dependent performance in Experiment 1 (*r*(14) = 0.235, *p* = 0.4, BF_10_= 0.441). Thus, we used the data of all observers in further analyses.

Data acquired from both days were converted to d′ scores according to Hacker and Ratcliff (1979). Unless otherwise stated, all analyses were carried out in MATLAB and JASP (JASP Team, 2022, JASP (0.15) for Windows). Mean performances for each condition and marginal means for each factor were reported as d′ scores with the associated standard errors. We chose the sample size for both experiments following Herwig and Schneider (2014), which yielded an observed statistical power of 0.849 for a one-way repeated-measures ANOVA. Bayesian factors (BFs) for main effects and posterior odds (POs) for post hoc comparisons were also provided for Experiment 1.

### Results

**Fig. 2:**
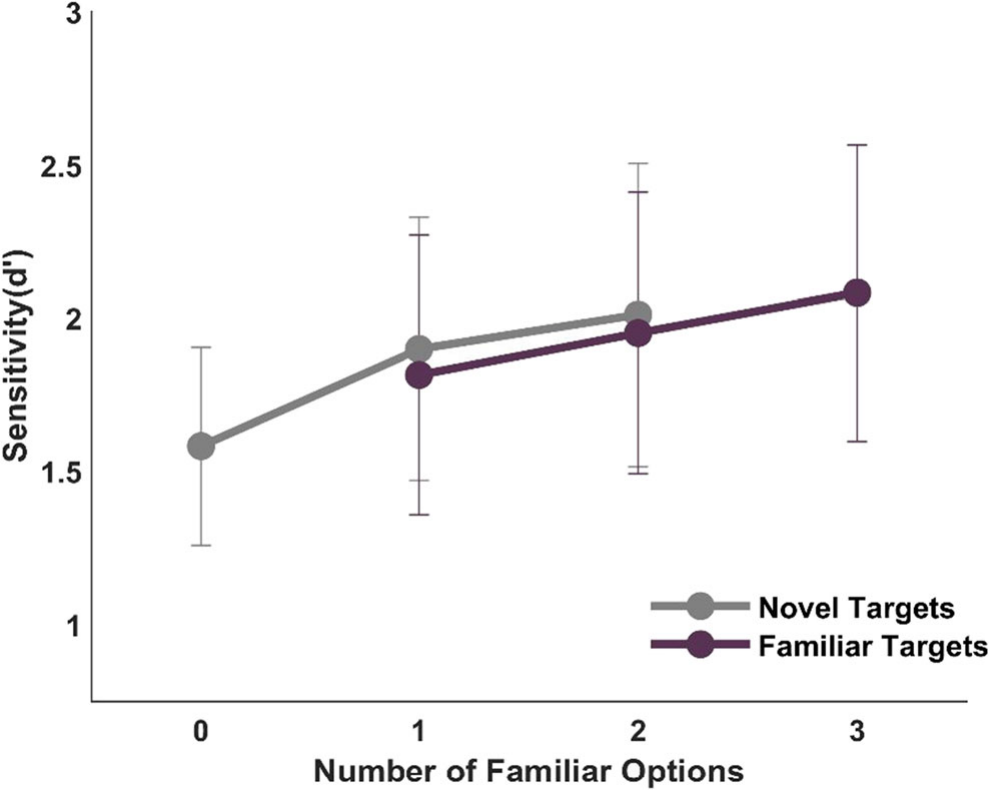
Mean performance of peripheral-foveal matching for familiar and novel peripheral targets across a different number of familiar foveal options. Error bars show 95% confidence intervals

On the first day, observers were familiarized with the designated greebles by completing a sham task without any instruction about the true purpose of the task. Then, they performed the memory check and the peripheral-foveal matching task. Figure 2 shows peripheral-foveal matching performances for familiar and novel peripheral targets across different number of familiar options. It can be seen that observers were able to perform the matching task even when the peripheral target and all matching options were novel. We first compared the performance in this condition to chance (d’ = 0) to confirm that peripheral-foveal predictions are not unique to familiar objects. A one-sample t-test yielded a significantly better than chance level performance in this condition (d′ = 1.58 ± 0.3, *t* (14) = 10.513, *p* < 0.001, *d* = .583, BF_10_ = 2.65 × 10^7^). Thus, observers were able to match targets that are only briefly seen on the periphery without needing the object-specific associations that familiar objects enjoy.

Figure 2 also shows that matching performance increased with increasing number of familiar options, hinting at the additional advantage of having familiar objects. Object-specific associations are suggested to be working bi-directionally to generate object-specific predictions. In other words, when peripheral or foveal information is present, a prediction is made for the counterpart information about how it would be like to see that item in the other viewing condition. We therefore analyzed trials with novel and familiar peripheral targets and with different number of familiar options separately to test the two different directions of predictions.

We first analyzed trials with novel peripheral targets and with none, one, or two familiar options (gray line in Fig. 2) to see how foveal-peripheral predictions from the familiar foveal options affect matching performance. A one-way repeated-measures ANOVA showed a significant effect of number of familiar foveal options (No familiar option: d′ = 1.58 ± 0.3, one familiar option: d′ = 1.9 ± 0.39 and two familiar options: d′ = 2.01 ± 0.45) on matching performance (*F*(2,28) = 5.686, *p* = 0.008, η^2^ = 0.289, BF_10_ = 5.536). Holm-Bonferroni-corrected post hoc comparisons indicated a significant performance improvement from no familiar option to one (*p* = 0.046, PO = 1.617) and two familiar options (*p* = 0.009, PO = 3.694).

Our design does not allow us to test peripheral-foveal prediction in isolation, since trials with familiar peripheral targets have at least one familiar option, which allow foveal-peripheral prediction. Therefore, we tested if the peripheral-foveal prediction from the familiar peripheral targets provides additional improvement to the matching performance by using trials with one or two familiar foveal options for familiar and novel peripheral targets (overlapping data points in Fig. 2). Thus, the additional advantage of peripheral-foveal predictions was tested by comparing performance in trials with novel peripheral targets with familiar peripheral targets with matched number of familiar options. A 2 (familiarity: Familiar: d′ = 1.88 ± 0.2, Novel: d′ = 1.95 ± 0.2) × 2 (Number of familiar choices: 1 familiar option: d′ = 1.86 ± 0.2, 2 familiar option: d′ = 1.98 ± 0.2) ANOVA yielded no significant difference for familiarity (*F*(1,14) = 0.523, *p* = 0.482, BF_10_ = 0.318), suggesting that there was no additional advantage of peripheral-foveal prediction. For completeness, we report the main effect of number of familiar options, which partially repeats the post hoc test comparing one familiar option to two familiar options, this time for the combination of familiar and novel peripheral target trials (*F*(1,14) = 1.473, *p* = 0.245, BF_10_ = 0.496). There was also no interaction between the two factors (*F*(1,14) = 0.013, *p* = 0.912, η^2^ = 0.001, BF_inc_ = 0.39).

We also analyzed separately the trials with familiar peripheral targets, for the sake of completeness. A one-way ANOVA suggested no significant effect of number of familiar options (1 option: d′ = 1.81 ± 0.42, 2 options: d′ = 1.95 ± 0.42, 3 options: d′ = 2.08 ± 0.44) on matching performance for familiar peripheral targets (*F*(2,28) = 1.59, *p* = 0.222, , BF_10_ = 0.476).

**Fig. 3:**
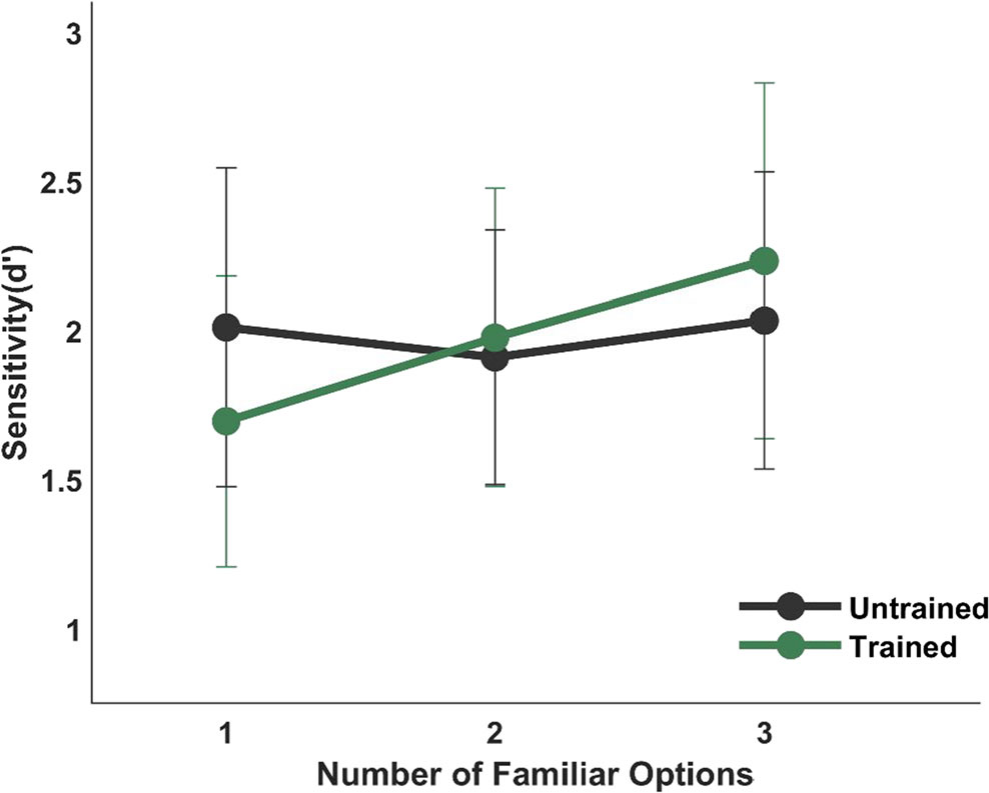
Mean peripheral-foveal matching performance for familiar targets at the trained (light green) or untrained (dark green) location across a different number of familiar options. Error bars show 95% confidence intervals

Location specificity of transsaccadic learning is an important question for understanding the role of transsaccadic predictions in everyday life. Previous studies have reported conflicting results on the location specificity of the transsaccadic learning (Herwig et al., 2018; Valsecchi et al., 2020; Valsecchi & Gegenfurtner, 2016). During training, visual targets were always presented on the left side of the screen. However, peripheral targets in Experiment 1 were randomly presented either on the left or on the right side of the screen with equal probability. Therefore, if object-specific predictions are dependent on learning location, we would expect the matching performance for familiar peripheral targets to be better when presented on the training location compared to the opposite location. Thus, we tested location specificity by comparing trials from Experiment 1, where the familiar peripheral target was presented either on the same or opposite side of the training stimuli (Fig. 3). A 2 (Location: Trained: d′ = 1.97 ± 0.21, Untrained: d′ = 1.99 ± 0.21) × 3 (Number of Familiar options: one option: d′ = 1.67 ± 0.22, two options: d′ = 1.48 ± 0.22, three options: d′ = 1.39 ± 0.22) Bayesian repeated-measure ANOVA comparing the location factor to the null model provided moderate evidence (*BF*_*10*_ = 0.217, *F*(2,28) = 0.019, *p* = 0.892) indicating that matching performance was independent from the training location. This is consistent with the findings by Valsecchi and Gegenfurtner (2016) and Valsecchi et al. (2020). As a sanity check, we also performed the same analysis on novel peripheral targets where we do not expect any effect of location. As expected, matching performance did not depend on the novel peripheral location (*F*(2,28) = 0.02, *p* = 0.892, BF_01_ = 3.54).

### Discussion

On the first day, observers familiarized with novel targets by executing saccades that are known to lead to stronger learning effects compared to passive viewing (Herwig & Schneider, 2014). The task of Experiment 1 was to match a briefly presented peripheral target with one of the three centrally presented options. We systematically varied the familiarity of the target and options. Our results show that peripheral-foveal matching is possible in the absence of object-specific predictions. After having confirmed this we returned to the main question of Experiment 1: Do object-specific predictions provide an additional advantage by means of bi-directional object-specific predictions (Herwig & Schneider, 2014)? That is, through association, peripheral sensory information could predict foveal information (peripheral-foveal prediction) as foveal sensory information could predict peripheral sensory information (foveal-peripheral prediction). Therefore, if observers benefit from peripheral-foveal predictions, we would expect better performance in trials with familiar peripheral targets than in trials with novel peripheral targets. Similarly, if observers benefit from foveal-peripheral predictions, we would expect performance to improve with increasing number of familiar options (in particular for novel peripheral targets). We observed an increase in peripheral matching performance with increasing number of familiar options for both familiar and novel peripheral targets. However, only the differences between no familiar option to one and two options for novel peripheral targets reached significance (Table 1). Given the high overall matching performance (~80%) in Experiment 1, the absence of a difference between one and two familiar options most likely represents a ceiling effect. Overall, having object-specific associations of familiar objects was advantageous when observers could compare the sensory information from the peripheral targets with the peripheral predictions from familiar foveal objects.

**Table 1:** Summary of hypotheses and associated results from Experiment 1. Each row shows which variables were used to test the given effect. Non-contributing variables are indicated with “-“

	Peripheral target	Number of familiar options	Location	*p*	*BF*_*10*_
Novel Object Prediction	Novel	0	-	< 0.001	2.65 × 10^7^
Foveal-Peripheral Prediction (Novel)	Novel	0, 1, 2	-	0.008	5.536
Peripheral-Foveal Prediction (Additional)	Novel, Familiar	-	-	0.482	0.318
Foveal-Peripheral Prediction (Familiar – Additional)	Familiar	1, 2, 3	-	0.222	0.476
Location Specificity	Familiar	-	Trained, Untrained	0.892	0.217

One explanation for foveal-peripheral predictions being more advantageous than peripheral-foveal predictions could be the higher reliability of the foveal information due to foveal vision having higher acuity and being less prone to crowding (for reviews, see Rosenholtz, 2016; Strasburger, 2020; Strasburger et al., 2011; Whitney & Levi, 2011). The difference in reliabilities could have been further amplified by our design since peripheral objects were briefly presented while foveal objects were presented until observers selected a response. Even if peripheral-foveal predictions provide an advantage for the peripheral-foveal matching task, our design does not allow testing for the isolated effect of peripheral-foveal prediction because trials with familiar targets had at least one familiar option. Thus, if there is any advantage of peripheral-foveal associations in peripheral-foveal matching, it is undermined by the effect of foveal-peripheral associations that are based on more reliable sensory information in our task.

Experiment 1 also provided evidence for spatial transfer of predictions from transsaccadic associations. The question of location specificity of transsaccadic learning is an important aspect for the ecological usefulness of familarity-based predictions. If transsaccadic associations of familiar objects were to be spatially bound, then the advantage of object-specific predictions would be highly specific and limited, as in some forms of perceptual learning (e.g., Karni & Sagi, 1991; for reviews, see Fahle, 2005; Fine & Jacobs, 2002; Goldstone, 1998; Watanabe & Sasaki, 2015). On the other hand, if the transsaccadic associations generalize then the visual system could use them for generating object-specific predictions to quickly identify and pre-process familiar peripheral objects in visual scenes. Previously, Valsecchi and Gegenfurtner (2016) and Valsecchi et al. (2020) provided evidence supporting the independence of transsaccadic learning for size, which is opposed by Herwig et al. (2018) showing specificity for spatial frequency. However, those studies used different simple visual features (size vs. spatial frequency) and eccentricities (opposite hemifield vs. various other locations) that can explain the contradictory results. In the current study, we used complex objects and compared matching performance in the trained and the mirrored location in the opposite hemifield to that in Valsecchi and Gegenfurtner (2016) and Valsecchi et al. (2020). Our results favor the spatial transfer of transsaccadic learning at least to the opposite hemifield. Together, the results of Experiment 1 suggest that the matching of peripheral and foveal information benefits from the spatially independent transsaccadic associations of familiar objects at least when foveal to peripheral predictions can be made.

## Experiment 2: Transsaccadic change detection

The first experiment showed that the presence of familiar foveal distractors improved the matching with novel peripheral targets. This implies that observers were able to detect the mismatch between the peripheral sensory information of the novel objects and the peripheral predictions of familiar distractors based on foveal sensory information. In the first experiment, observers were required to match peripheral and foveal objects when they were not allowed to execute saccades to alter the retinal location of the objects. However, in daily life we constantly make saccades to peripheral objects to obtain foveal information that offers better resolution. By executing a saccade, the visual system integrates pre- and post-saccadic information related to saccade targets as well as to other attended locations (Schut et al., 2018; Stewart & Schütz, 2019a; Stewart & Schütz, 2019b). However, if there is a high discrepancy between pre- and post-saccadic objects, then the visual system segregates this information (e.g., Atsma et al., 2016; Demeyer et al., 2010; Tas et al., 2012; Tas et al., 2021). Peripheral-foveal associations should provide richer object-specific predictions compared to generative predictions of novel objects that can further facilitate the detection of discrepancies between pre- and post-saccadic information. Therefore, in the second study we hypothesized that transsaccadic changes would be more detectable in familiar objects than in novel objects.

Transsaccadic change detection of location (Deubel et al., 1996; Poth et al., 2015) and features (Hübner & Schütz, 2021; Weiß et al., 2015) is known to be improved by a blank period introduced after saccades. Atsma et al. (2016) suggested that the visual system retains both peripheral and foveal information to arbitrate between integration or segregation of transsaccadic information. If there is a high discrepancy between the two types of information or a blank, which suggests a violation of the stable world assumption (Deubel et al., 1996), the visual system segregates the foveal and peripheral information. As a result, the transsaccadic change becomes more apparent to the observer. Within the framework of the transsaccadic feature prediction mechanism, we consider two possibilities for how the presence of a blank could differentially improve transsaccadic change detection of familiar and novel objects. On the one hand, blanking could be more effective at improving the change detection in novel stimuli since detecting changes in familiar stimuli should already be facilitated by the peripheral-foveal associations. On the other hand, blanking could improve the change detection performance for familiar stimuli since the separation of information by blanking would help to segregate more precise object-specific predictions from the sensory information of the postsaccadic stimulus.

To test whether peripheral-foveal prediction of familiar objects improves transsaccadic change detection and how blanking contributes to the detection of changes in familiar and novel stimuli, we conducted a second experiment. Since we used complex objects that varied in high level features, we varied the magnitude of change (*change difficulty* here after) as a control for large discrepancies between transsaccadic objects that could lead to segregation of transsaccadic information.

### Methods

#### Observers

In total, 18 new observers (13 females, mean age = 23.83 ± 3.07 years) with normal or corrected-to-normal vision participated to the experiment. Observers were university students; they signed an informed consent form and were compensated with 8 euros per hour.

#### Stimuli and apparatus

The stimuli and apparatus were identical to the training except for the following changes. The same four familiarized greebles from the training phase and eight novel greebles were used. For each of the 12 greebles, a hard change greeble and an easy change greeble that vary in one or two dimensions, respectively, were chosen to serve in change trials.

#### Procedure

The observers underwent the same training and memory check procedure as the observers in the first experiment. After the memory check, observers proceeded with the transsaccadic change detection task. Each trial started with a fixation period of 500–1,500 ms. While fixation was maintained, a presaccadic peripheral target was presented either 10° left or right of the fixation target. Saccades were detected online when the horizontal gaze position traveled more than 4° from the target. The detection of the saccade triggered the exchange of the presaccadic target with a different (novel) or an identical postsaccadic target. For the blank trials, the presaccadic target was extinguished and exchanged with the postsaccadic target only after a 300-ms blank period. In all conditions, the postsaccadic target was presented for 300 ms. The task of the observer was to report whether the pre- and post-saccadic target were identical or not. Each observer completed blank trials and no-blank trials in separate blocks. The order of the blank and no-blank blocks was counterbalanced by reversing the order for every other observer. Typically, blank and no-blank trials are interleaved in this paradigm, but we decided to block these conditions to avoid temporal variation in the onset of the postsaccadic target and to maximize the consistency of trials within a block. The viewing condition (familiar target viewed peripherally or foveally) and the difficulty (hard: one different feature; easy: two different features) of the target was manipulated across observers in the following way: Half of the observers completed the task where familiar stimuli were presented as a postsaccadic target for hard change trials and as a presaccadic target for easy change trials. For the other half of the observers the position of the familiar stimuli was switched for easy and hard change trials.

#### Data analysis

Similar to Experiment 1, we intended to use the memory check as a control measure for familiarization training. To asses if the memory check was an adequate measure of observers’ familiarity for Experiment 2, we checked the relationship between performance in the memory check and the change detection task. We correlated the memory performance with the difference between novel and familiar objects in no-blank conditions, separately for easy and hard change trials. There was no significant relationship between memory check performance and easy change trials (*r*(17) = 0.171, *p* = 0.49, BF_10_= 0.361) or hard change trials (*r*(17) = -0.158, *p* = 0.53, BF_10_= 0.35) . Thus, we did not exclude any observers on the basis of their memory check performance.

As in the first experiment, the data acquired during the training phase were converted to d′ scores. The change difficulty manipulation for the transsaccadic change detection task does not allow us to make a direct d′ calculation since the signal-absent (same) trials cannot be assigned to signal-present (different) trials with two difficulties. Therefore, the data acquired from the experiment phase were converted to d′ using the revised d′ table from Hacker and Ratcliff (1979). We compared main effects to the null model for obtaining BFs. For interactions, we added main effects to the null model to calculate BFs. Posterior odds were reported for post hoc tests.

Observers were separately tested in two viewing groups where the order of the exchanged stimulus for the familiar stimulus trials was switched across the difficulty conditions. By means of a one-way mixed ANOVA, we first compared the two viewing groups using familiar trials for the blanking and no-blanking conditions. There was no significant difference between the two viewing groups (*F*(1,16) = 0.691, *p* = 0.418, BF_10_ = 0.378). Therefore, we merged the data from the groups for the subsequent analyses. In the merged data, trials with at least one familiar stimulus were assigned to the familiar trial group regardless of change difficulty or being pre- or post-saccadic. Observed statistical power for the all main effects and the interaction from a 2 × 2 × 2 ANOVA was larger than 0.8.

### Results

**Fig. 4:**
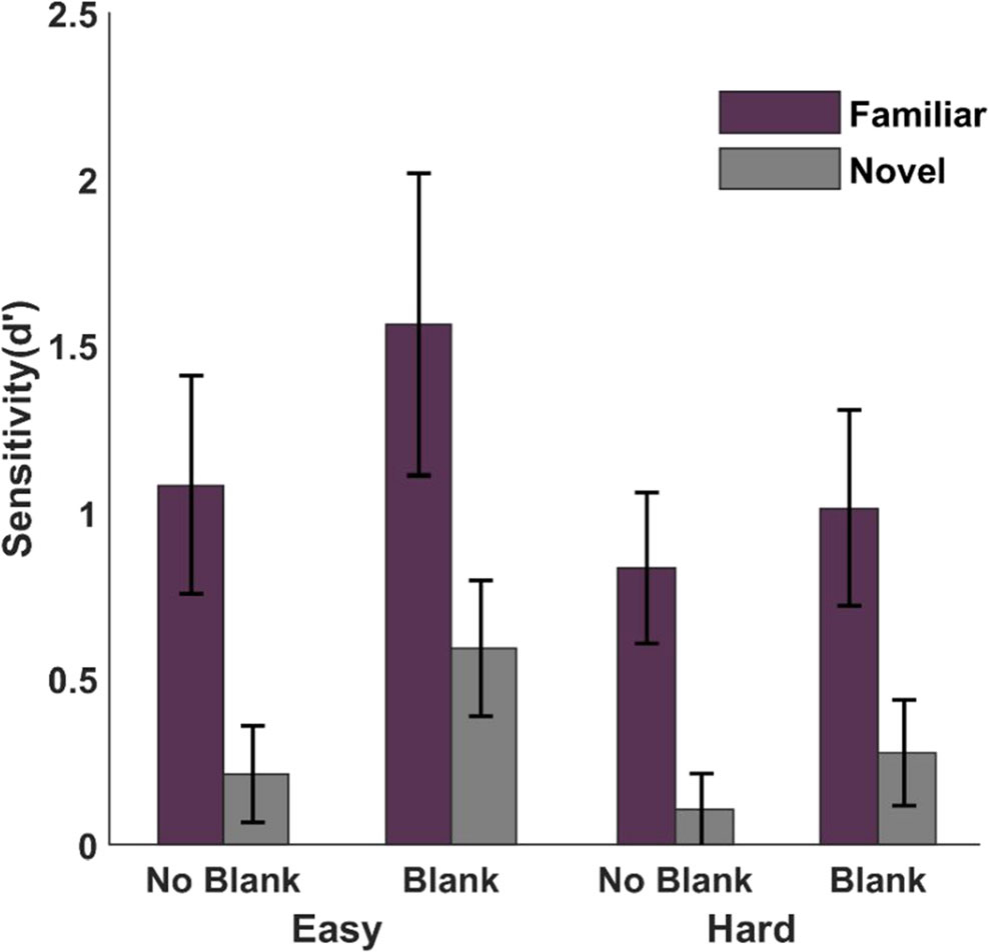
Mean change detection performance with or without blank for familiar (purple) and novel (gray) stimuli across change difficulty (**left panel:** easy change; **right panel:** hard change). Error bars show 95% confidence intervals

Similar to Experiment 1, observers in Experiment 2 were familiarized with the training stimuli on day 1 then proceeded to the test phase. We conducted a 2 (Familiarity: Familiar, Novel) × 2 (Blanking: Blank, No-blank) × 2 (Change Difficulty: Easy, Hard) repeated-measures ANOVA (Fig. 4*)*. There was a main effect of familiarity (*F*(1,17) = 27.101, *p* < 0.001, η^2^ = 0.061, , BF_10_ = 7.81) indicating a better change detection performance for familiar (d′ = 0.86 ± 0.08) than for novel (d′ = 0.56 ± 0.08) stimuli. Similarly, change detection performance improved with the presence of a blanking period (F(1,17) = 10.084, *p* = 0.006, η^2^ = 0.06, , BF_10_ = 7.08, blank: 0.86 ± 0.09 vs. no blank: 0.56 ± 0.09). As expected, the change difficulty also modulated the performance (*F*(1,17) = 49.156, *p* < 0.001, η^2^ = 0.447, , BF_10_ = 1.13 × 10^15^, easy: 1.22 ± 0.01 vs. hard: 0.3 ± 0.01). Change difficulty did not interact with blanking (*F*(1,17) = 0.144, *p* = 0.709, BF_inc_ = 0.286), familiarity (*F*(1,17) = 3.676, *p* = 0.072, BF_inc_ = 0.977), or a combination of the two (*F*(1,17) = 0.365, *p* = 0.554, BF_inc_ = 0.171), which suggests that the difficulty manipulation was balanced for familiar and novel stimuli.

However, we did find a significant interaction between blanking and familiarity (*F*(1,17) = 10.444, *p* = 0.005, η^2^ = 0.011, BF_inc_ = 2.152). To check if familiar and novel objects benefitted from blanking to a different degree, we compared the difference between blank and no-blank conditions for familiar and novel conditions. Since the change difficulty could modulate the blanking effect, we analyzed easy and hard change trials separately. We calculated blanking effects for familiar and novel conditions by subtracting the performance in blank conditions from no-blank conditions for easy and hard change trials. We found that familiar objects benefitted from blanking more than novel objects in both easy (*t*(17) = 2.361, *p* = 0.03, *d* = 0.556, BF_10_ = 2.13) and hard change conditions (*t*(17) = 2.747, *p* = 0.014, *d* = 0.647, BF_10_ = 4.06).

Finally, unlike Experiment 1, Experiment 2 allowed us to test the isolated effect of peripheral-foveal predictions. The design of Experiment 1 only allowed us to test the additional contribution of peripheral-foveal predictions since for familiar peripheral targets there was always a familiar foveal option that could provide foveal-peripheral predictions. However, in Experiment 2, this can be directly tested by comparing the familiar presaccadic stimulus trials to the novel stimulus trials. Since change difficulty of these trials was manipulated across groups, for one group these trials were hard changes and for the other group they were easy change trials. We controlled change difficulty by comparing familiar presaccadic trials with novel trials with the same change difficulty. Therefore, if the familiar presaccadic stimulus trial was also a hard change trial, the performance of the observer was paired with their performance in hard change novel trials. By matching the change difficulty of familiar and novel trials, we tested whether peripheral-foveal predictions provide extra benefit in change detection. A 2 (peripheral object: familiar, novel) × 2 (blanking: blank, no blank) repeated-measures ANOVA comparing trials with familiar and novel peripheral targets (with matched change difficulty) yielded a significant main effect of peripheral object familiarity (*F*(17,1) = 12.614, *p* = 0.002, η^2^ = 0.135, BF_10_ = 7.023, d′ =1.02 ± 0.14 vs d′ =0.66 ± 0.14) on change detection performance. As expected, there was also an effect of blanking (*F*(17,1) = 5.204, *p* = 0.036, η^2^ = 0.129, BF_10_ = 6.054, d′ =1.01 ± 0.15 vs d′ =0.68 ± 0.15) and interaction (*F*(17,1) = 5.914, *p* = 0.026, η^2^ = 0.034 BF_inc_ = 0.812).

### Discussion

In Experiment 2, we asked observers to report changes to presaccadic objects during saccades. In line with our hypothesis, the results showed that transsaccadic changes to familiar objects were more readily detectable compared to novel objects especially when they were blanked (Table 2.).

**Table 2.** Summary of the hypotheses and associated results from Experiment 2. Each row shows which variables were used to test the given effect. Non-contributing variables are indicated with “-“

	Familiarity	Blanking	*p*	*BF*_*10*_
Familiarity	Novel, Familiar	-	< 0.001	7.81
Blanking	-	Blank, No blank	0.006	7.08
Familiarity x Blanking	Familiar, Novel	Blank, No blank	*easy*: 0.03 *hard*: 0.014	2.13 4.06
Peripheral-Foveal (Familiar)	Novel, Familiar (saccade targets)	-	0.002	7.023

The familiarity effect that we observed can be well explained by the framework suggested by Atsma et al. (2016). They argued that the visual system holds on to the peripheral and foveal information to arbitrate integration or segregation of information based on the degree of discrepancy between the two types of information. The more precise object-specific predictions for familiar objects provide an advantage in detecting transsaccadic changes. Therefore, when the visual system evaluates the discrepancy between a familiar object with another object, it can compare the object-specific transsaccadic prediction with the sensory information of the other object. However, any transsaccadic exchange between two novel objects provides less precise predictions and thereby makes the change detection harder.

Another way of improving the detection of transsaccadic changes is to introduce a blank period before the transsaccadic change (e.g., Deubel et al., 1996; Hübner & Schütz, 2021; Weiß et al., 2015). We found that familiar objects benefitted more than novel objects regardless of change difficulty. In other words, the separation of pre- and post-saccadic information by blanking was more beneficial for detecting changes in familiar objects compared to novel objects. One reason could be that the object-specific predictions are informative for the visual system but were masked by the postsaccadic input, while general predictions generated for novel stimuli were also masked but not as informative as object-specific predictions to improve the change detection. Therefore, removing the postsaccadic masking or overwriting by means of blanking (Grzeczkowski et al., 2020; Tas et al., 2021) might have amplified the difference between the two types of prediction.

Finally, we tested if peripheral-foveal predictions from transsaccadic associations can be effective at signaling transsaccadic changes. Experiment 1 showed that the foveal-peripheral predictions were largely responsible for the familiarity effect that we observed in the peripheral-foveal matching task. However, the design of Experiment 1 did not allow comparison of the isolated effects of peripheral-foveal since familiar peripheral target trials always contained familiar options. This comparison can be made by analyzing the trials with peripheral familiar targets in Experiment 2. Our results showed that, at least for the transsaccadic object change detection, having peripheral-foveal predictions was advantageous. We argue that the different pattern of results in the two experiments could be explained by the differences in the relative reliability and usefulness of information in the two tasks. Although peripheral and foveal predictions were useful for both tasks, only in the change detection were both predictions equally informative. On the one hand, in Experiment 1, observers had one source of peripheral-foveal prediction with time-constrained sensory information and three sources of foveal-peripheral prediction without any time constraints on the sensory information. Therefore, the visual system had multiple sources of information to perform the task. Thus, the foveal-peripheral predictions could have been weighted more by having more reliable sensory information. In contrast, the availability of peripheral and foveal information was more balanced in Experiment 2, making the peripheral-foveal prediction more informative compared to Experiment 1. Thus, we argue that object-specific predictions work bi-directionally but the prediction in each direction is used based on their reliability and relevance for the task.

## General discussion

Through the course of life, the visual system samples information with peripheral and foveal vision. Each saccade directed to an object provides peripheral information and foveal information about the targeted object. The visual system uses the available sensory information to make transsaccadic predictions about how an object would look like in different viewing conditions (e.g., Herwig & Schneider, 2014; for reviews, see Huber-Huber et al., 2021; Stewart et al., 2020).

We argue that object predictions can be made at two levels. First, object predictions can be made based on a generative model about how object appearance changes at different eccentricities without requiring object-specific learning. Second, a more precise prediction can be made based on the previously associated peripheral and foveal information belonging to the same object, as suggested by Herwig and Schneider (2014). We argue that object predictions can be conceptualized as a ladder where at the lower end coarse predictions can be made for both novel and familiar objects based on a generative model about their appearance at different eccentricities and at the upper end more precise object-specific predictions can be made for familiar objects from previously associated peripheral and foveal object information. In this study, we investigated if object-specific predictions for familiar objects provide an advantage in peripheral-foveal matching and transsaccadic change detection tasks compared to novel objects, which lack these predictions. Our results suggest that while more general predictions are sufficient to perform peripheral-foveal matching and change detection tasks with novel objects, familiar objects with more precise predictions had a significant advantage over novel objects on both tasks. Previous studies have shown that these predictions work bi-directionally to support various visual tasks such as peripheral recognition or visual search (Herwig & Schneider, 2014; Osterbrink & Herwig, 2021). The design of Experiment 1 allowed us to systematically manipulate the familiar object information available to peripheral and central vision. We found that observers benefitted more from foveal-peripheral predictions. In addition, the availability of peripheral-foveal predictions was advantageous in Experiment 2. Therefore, our results not only support bi-directionality of object-specific predictions, but also show how task demands and available information could influence the direction of prediction.

Object-specific predictions that can be made for familiar objects could be decisive when performing demanding visual tasks such as medical image interpretation (e.g., Bertram et al., 2013; Lago et al., 2021; Williams et al., 2021). The association of transsaccadic information for familiar objects could be an important factor in visual expertise and everyday life, even for very overtrained stimuli, such as faces. Familiar faces are processed more rapidly and with less attention compared to novel faces (Gobbini et al., 2013). Furthermore, face processing benefits from transsaccadic effects such as the preview benefit (Buonocore et al., 2020; Huber-Huber et al., 2019). Moreover, Osterbrink and Herwig (2021) showed that transsaccadic predictions can be made for faces and that they bias judgments towards the acquired peripheral-foveal associations. Following our ladder metaphor, the visual system can generate predictions about how faces look like in different viewing conditions when we encounter a stranger. When we encounter the same face again or start to become more acquainted with it, in addition to our first general predictions, the visual system can make predictions specific to that face. By having these more precise predictions, we can now more easily recognize the face or detect changes to it.

The transsaccadic predictions for the pre- and post-saccadic information can also help to arbitrate between integration and segregation of pre- and post-saccadic information (Atsma et al., 2016). Previous studies have shown that the visual system is sensitive to transsaccadic changes to objects and could adapt itself within a brief period (Cox et al., 2005; Valsecchi et al., 2020; Valsecchi & Gegenfurtner, 2016). As a result, the visual system builds transsaccadic associations of peripheral and foveal information reflecting the change caused by adaptation (Cox et al., 2005; Herwig et al., 2018; Herwig & Schneider, 2014; Köller et al., 2020; Osterbrink & Herwig, 2021; Paeye et al., 2018; Valsecchi et al., 2020; Valsecchi & Gegenfurtner, 2016) or learning as in the current experiments. Previous studies showed that pre- and post-saccadic information is integrated based on their reliabilities (e.g., Ganmor et al., 2015; Hübner & Schütz, 2017; Stewart & Schütz, 2019a; Wolf & Schütz, 2015). However, when the saccade target is drastically changed beyond adaptation, for instance when it is exchanged with another object (Demeyer et al., 2010; Tas et al., 2012; Tas et al., 2021), the presaccadic information is not integrated or overwritten by the postsaccadic information. The object-specific predictions could further help the visual system to arbitrate between perceptual continuity and discontinuity. Observers in Experiment 2 were better at detecting changes to familiar objects than novel objects. Thus, object-specific transsaccadic predictions are more informative than their more general counterparts at detecting changes.

In conclusion, we argue that general transsaccadic predictions can be based on a generative model of typical differences in appearance at different eccentricities and that more precise object-specific predictions can be acquired by transsaccadic learning. While the visual system can perform object matching and change detection using general predictions, it specializes in familiar objects by directly associating peripheral and foveal information for better precision. The higher precision provided by object-specific predictions of familiar objects could be supporting visual expertise as well as help the visual system to detect changes in the environment more readily.

## Data Availability

The original stimulus images are courtesy of Michael J. Tarr, Carnegie Mellon University, http://www.tarrlab.org/ Data generated during experiments can be found at 10.5281/zenodo.7520182. None of the experiments was preregistered.
